# Goal-Directed Resilience in Training (GRIT): A Biopsychosocial Model of Self-Regulation, Executive Functions, and Personal Growth (*Eudaimonia*) in Evocative Contexts of PTSD, Obesity, and Chronic Pain

**DOI:** 10.3390/bs5020264

**Published:** 2015-06-01

**Authors:** Martha Kent, Crystal T. Rivers, Glenda Wrenn

**Affiliations:** 1 Research Department R151, Phoenix VA Health Care System, 650 E. Indian School Rd., Phoenix, AZ 85012, USA; 2 Department of Psychology, Arizona State University, 651 E. University Drive, Tempe, AZ 85287-1104, USA; E-Mail: ctrivers@asu.edu; 3 Department of Psychiatry and Behavioral Science, Morehouse School of Medicine, 720 Westview Dr. SW, Atlanta, GA 30310, USA; E-Mail: gwrenn@msm.edu

**Keywords:** PTSD, interventions, resilience, coping, adaptation, Goal-Directed *vs.* stimulus-based, pain, prevention, Veterans

## Abstract

This paper presents a biopsychosocial model of self-regulation, executive functions, and personal growth that we have applied to Goal-Directed Resilience in Training (GRIT) interventions for posttraumatic stress disorder (PTSD), obesity, and chronic pain. Implications of the training for the prevention of maladaptation, including psychological distress and health declines, and for promoting healthy development are addressed. Existing models of attention, cognition, and physiology were sourced in combination with qualitative study findings in developing this resilience skills intervention. We used qualitative methods to uncover life skills that are most salient in cases of extreme adversity, finding that goal-directed actions that reflected an individual’s values and common humanity with others created a context-independent domain that could compensate for the effects of adversity. The efficacy of the resilience skills intervention for promoting positive emotion, enhancing neurocognitive capacities, and reducing symptoms was investigated in a randomized controlled trial with a veteran population diagnosed with PTSD. The intervention had low attrition (8%) and demonstrated improvement on symptom and wellbeing outcomes, indicating that the intervention may be efficacious for PTSD and that it taps into those mechanisms which the intervention was designed to address. Feasibility studies for groups with comorbid diagnoses, such as chronic pain and PTSD, also showed positive results, leading to the application of the GRIT intervention to other evocative contexts such as obesity and chronic pain.

*The experiences of camp life show that man does have a choice of action…become a plaything of circumstances…or striv(e) and struggl(e) for a worthwhile goal…*

*Viktor E. Frankl* 

## 1. Introduction

Extreme situations expose behaviors that sustain life and challenge adaptation. It is in this crucible that the adaptive fight-flight-freeze responses of mammals become visible. A century of research has examined the physiological mechanisms of fight-flight, associated homeostatic regulation, and behavioral responses [1], and in recent decades has tied homeostatic dysregulation, hyperarousal, and behavioral symptoms to traumatic experiences [2]. The dual relationship between goal-directed action and stimulus-based responses, two processes that motivate action selection, has emerged independently in a number of research areas, with these two distinct modes of responding most clearly reflected in extreme situations where people’s exceptional survival (*i.e.*, cases where people experience growth and find meaning) is linked to goal-directed action while traumatic stress is typified by the more reactive stimulus-based response. Goal-directed action is more psychological in nature, and is motivated by belief in a contingency between an outcome and a goal and a desire for an outcome [3]. By contrast, the stimulus-based response is an automatic response that may be physiological, as in fight-flight-freeze during extreme situations, or habitual, as with actions that have become associated with events through learned responses [4]. Goal-directed action, an executive function, is compromised and displaced by symptoms of traumatic stress [5,6].

Can reactive responding be reversed for people suffering from posttraumatic stress disorder (PTSD) in favor of goal-directed, positive adaptation? This central question has driven our intervention research and provides a guidepost for this paper. We introduce a revised resilience skills building intervention for contexts which we identify as “evocative”, such as the experience of threatening contexts in trauma, stimulus salience of obesity, and interoceptive pain in chronic pain. We identify the more specific element of goal-directedness in resilience training and theoretically ground it in an interdisciplinary body of empirical research and our findings from qualitative and intervention pilot data. Our goals in the paper are to: (1) review models proposing versions of goal-directed *versus* stimulus-based responding from disparate areas of research; (2) describe the revised Goal-Directed Resilience in Training (GRIT) method for restoring goal-directed action in individuals with reactive adaptations to extreme contexts; (3) propose a model for implementing the goal-directed intervention in diverse evocative contexts; (4) report on preliminary tests of the model with several populations; and (5) discuss its implications for intervention approaches and for prevention.

It is helpful to begin this discussion by specifying that we frame goal-directed action as a mechanism by which people living through extreme situations may nevertheless experience resilient outcomes. Resilience is defined here as positive adaptation to evocative contexts; this is consistent with the prevailing definition of resilience as requiring a positive adaptation in response to a stressor [7,8]. The recognition of resilience as an adaptive response is relatively recent, beginning with the studies in the 1970’s of children growing up in adverse circumstances without showing symptoms of psychopathology [9,10]. Strong evidence suggests that positive social relationships and effective actions in the environment, such as school achievement, may be protective factors for children. The study of adult resilience is much more recent. It has modeled the different trajectories people take following difficult life events (e.g., [11]) and has tackled issues typical of an emerging field: definitions, methodological issues, or the measurement of resilience as process and/or outcome (e.g., [9,12,13]). Our own work shows that resilience responses in evocative contexts are most likely to be those that are goal-directed, intentional, prospective, future-oriented actions/emotions that originate with the individual (for overview see: [2,14]). Through this work, we have identified approach/engagement behaviors and social relatedness as two main features of adult adaptive responses to extreme situations. These qualities contrast with the withdrawal/defense of fight-flight or the reactive symptoms of PTSD, a dichotomy that belongs to a long tradition of approach-avoidance concepts [15]. We will next review examples of approach-avoidance constructs, the emergence of goal-directed *versus* stimulus based responding, and their relevance to resilience and the training model proposed in this review.

### 1.1. Approach/Engagement *versus* Withdrawal/Defense

The concepts of approach-avoidance and their variants are actually fairly common and appear in a variety of diverse research literatures, originating at times independently of one another. Examples are: physiologically relevant research, most notably the sympathetic and parasympathetic responses [1]; action research that distinguishes voluntary action from stimulus-based action [16], attention research that proposes goal-directed *versus* stimulus-driven attention [17]; positive psychology research investigating eudaimonia *versus* hedonia [18]; and social learning concepts such as locus of control [19] and self-efficacy [20]. Some of the most innovative research includes the development of contemporary models of predictive and reactive biobehavioral control [21,22,23], interactions of large-scale brain networks involving the salience network and the default mode [24], genomic findings related to pleasure and eudaimonia of Fredrickson and colleagues [25], and research on the role of emotions in event coding by Gentsch and Synofzik [26].

#### 1.1.1. Motivational Models: Approach-Avoidance

Historical examination of the approach-avoidance dichotomy [27] rank it among the oldest and most enduring explanatory concepts describing the nature of living organisms. The ancient Greek hedonist philosophers prescribed the pursuit of pleasure and the avoidance of pain as the key ingredients for a good life. The concepts have survived well to the present day (for examples see: [28,29,30,31,32,33]), as is evident in the special section of *Emotion Review* [34] devoted to approach-avoidance motivation and emotion. These contemporary contributors agree that normal adaptation entails appetitive physical and psychological orientation toward reward and incentive and an aversive physical and psychological orientation away from punishment and threat [34].

It is noteworthy that current work continues to define approach-avoidance motivation in terms of reward and punishment. The core evaluative dimension of positive/negative valence still appraises stimuli as beneficial or harmful and is seen as evolutionary engrained evaluation that supports biological adaptation. These contemporary positions are rooted in earlier treatments, such as the work of Schneirla, who recognized centrality of the biological and evolutionary nature of approach-avoidance and extended these to other life forms and identified mechanisms of stimulus intensity and energy where low intensity signified approach and brought benefits while high intensity signified harm or death [35]. The dimensions of valence, intensity, and direction of behavior as essential features of motivation has continued to the present day (see the special section of *Emotion Review* [34]). In addition, research has also evolved to recognize how multifaceted motivation is and that a main adaptive feature is its flexibility. In simple one-celled organisms approach-avoidance is simple and rigid but in complex organisms it is much more intricate and flexible. An important abiding mechanism of motivation is stimulus evaluation and the recognition that it is also multifaceted and can range from subcortical physical reflexes to higher-order cortical processes [36]. The recent contributions of research on goals to understanding the complexity of motivation are summarized in two recent volumes [37,38].

#### 1.1.2. Emotional Models: Approach-Avoidance

Drawing on Darwin, Ekman [39] represented the view of emotions as discrete categories rather than dimensions. His work provided extensive physiological, neural, and cross-cultural evidence in support of the notion that facial expressions were directly associated with felt emotion and, thus, primarily convey emotions. Evolutionary processes had endowed each emotion with a different adaptive function that activated different brain regions. By contrast, dimensional approaches held that emotions were not discrete and separate, but differ only in degree along one or more dimension.

In factor analytic studies, theories of emotion attribution [40,41] have produced two-dimensional models that capture other variations of approach-avoidance. Here investigators asked for judgments about emotional states and emotional objects, using emotion terms. The goal was to identify the basic features of emotions. Results uncovered fundamental conceptual dimensions, the most common being two-dimensional ones of pleasant *versus* unpleasant and activated *versus* deactivated dimensions e.g., [40,42,43]. Subsequent investigations confirmed the two-dimensional structure, with various modifications [41]. Concepts of positive and negative affect have become more nuanced and seen as independent affective systems that can be experienced concurrently but can become bipolar in extreme situations where they “collapse into a single bipolar dimension with highly inversely coupled affect” [44]. These findings are applicable to goal-directed action and stimulus-based responding in that they most likely are also independent systems but become bipolar in extreme environments.

Gray [45,46] proposed two general systems as the basis of behavior and affect, namely the behavioral inhibition system (BIS) that inhibits behavior leading to aversive outcomes and the behavioral activation system (BAS) of behavior that leads to reward. In general, BIS promoted vigilant scanning for threat while BAS was seen as an appetitive system of approach to pleasant and rewarded results. Support for the neurobiological basis for the BIS and BAS systems came from such work as that of Sutton and Davidson [47], who related hemispheric asymmetry to the BIS and BAS systems. Concepts of behavioral activation and inhibition have ascended especially with imaging studies of reciprocal cortical networks, such as the salience network and default mode [24], that are not specifically tied to reward-punishment processes. Reciprocal activation-inhibition are processes that likely underpin action *versus* stimulus-based responding.

#### 1.1.3. Action Models: Intention-Based *versus* Stimulus-Based Responding

The ideomotor approach to action has sidestepped the powerful dualities of reward-punishment or negative-positive valence by focusing on the consequences of actions and their representation. Prinz [48], Hommel and colleagues [49,50] proposed that the representation of action and the motor action share common coding mechanisms that allow action to be goal-directed and thereby respond to the outcomes of the person’s own actions rather than to environmental stimuli. It is, therefore, anticipated future outcomes instead of environmental stimuli that determine behavior [51].

Intention-based and stimulus-based actions have been the subject of action research [16,52,53], which has identified self-initiated actions that involve brain mechanisms and cognitive processes distinctly different from reflexive or responsive movement [54]. The conscious intention to act originates with *preparation for action*, which differs from the reaction to a stimulus that demands a certain response.

Broadly conceived, humans have two types of responses to the environment: They act to achieve results in the environment or they accommodate to the demands of the environment. For example, Herwig and Waszak [52] draw the contrast between the intentional act of pressing a cappuccino button on a dispenser for a cup of coffee *versus* the reflexive response of stepping on the brakes at a red traffic light. According to the ideomotor position, intention-based actions are directed by the effects that follow actions. However, stimulus-based actions depend on stimuli that precede actions, such as the traffic light—a stimulus that does not result from a person’s action. What holds action-effect association and stimulus-response association in place is the decreased time interval in each case. These have come to be known as *action-effect binding* and *stimulus-response binding* respectively. Although the main experimental paradigm used to investigate intention-based and stimulus-based action rely on very abstract reaction time studies, the findings are striking in having identified these two basic modes of responding and the neural processes supporting each.

#### 1.1.4. Attention Models: Goal-Directed *versus* Stimulus-Driven

Attention is another area that has not come under the spell of the dualities of reward and valence. The new dichotomy arising in the action research reviewed briefly above has its parallel in attention studies of goal-directed *versus* stimulus-driven attention constructs. In their classical meta-analysis of fMRI studies on attention, Corbetta and Shulman [17] identified neural systems for voluntary control of attention or *endogenous* control and automatic reflexive control or *exogenous* control. The endogenous control involves goal-directed (top-down) voluntary control of attention. It integrates knowledge, expectations, and goals in voluntary decisions to pursue goals. It is supported by the dorsolateral prefrontal cortex (PFC) involved in selecting stimuli and responses. In exogenous control, external objects or events claim a subject’s attention. It specializes in detecting salient or unexpected stimuli that stand out in the environment and that capture attention in automatic reflexive ways. Exogenous control is supported by the ventral frontoparietal network, a network that can work as a circuit breaker for the dorsal system, directing attention to the salient events and, thus, away from goal-directed tasks.

As with other dual systems approaches, Corbetta and Schulman’s work has evolved to become the “reorienting system” of the brain, a system that can change an ongoing course of action and respond to threat or to any advantages present in the environment [55]. Corbetta and Schulman’s 2002 [17] paper has had a powerful impact on attention research, being the most frequently cited paper of the decade in a survey of publications by *Nature Reviews Neuroscience*, notwithstanding the nonexistence of attention asserted by Anderson [56] whose claim for attention as an emergent property has not caught on.

The success of the dual attentional processes described above has few parallels in the clinical literature Here clinical research questions are framed in terms of the effects trauma has on various processes, not making room for the emergence of broader and more fundamental questions. For example, Morey and colleagues [57] investigated how trauma-related information diverted attention in (PTSD) and how trauma-related environmental cues modulated working memory networks. They found that the PTSD group showed greater activation for combat related *versus* non-combat distractors in the ventral emotion processing regions. The PTSD group also showed a more generalized PFC disruption to both combat and non-combat task-irrelevant distractors and performed more poorly in distractor scenes. This PTSD research, with its emphasis on what trauma does rather than on what of normal life is lost and needs to be regained, does not tie the findings to the broader disruption of positive goal-directed functioning central to a normal life. When the focus is on the pathology of trauma, it becomes easy to get caught up in etiology while losing sight of mechanisms suggested by the losses in normal life. This narrow focus may account for the relative absence of research on goal-directed action in clinical research.

#### 1.1.5. Brain Functions: Predictive and Reactive Biobehavioral Programs

A number of cortical models are relevant to goal-directed action and stimulus-based responding central to this paper, such as the work on intention and reactivity by Astor-Jack and Haggard [58] on the activation of the motor system either by a person’s intentions or by external stimuli, and the work of Hannus and colleagues [59] on stimulus-driven and user-driven control of visual attention. We will describe briefly the predictive and reactive model proposed by Tops and colleagues [21,22,23]. They connect prediction and reactivity to the environment, defining them as two basic biobehavioral programs that are adapted to control behavior most effectively in different types of environments, summarized in the predictive and reactive control systems (PARCS). Thus, the reactive program is most adaptive in low predictable environments while the predictive program is most effective in highly predictable environments. The reactive program is characterized by fast associative learning where the environment requires a quick response and behavior is guided by momentary feedback from the environment. Attention is focused on stimuli that are urgent and close in time and space. The stimuli can be negative or positive, such as avoiding a threat or getting a reward. By contrast, the predictive program is specialized for guiding behavior that is formed and kept stable by slow learning. There is less urgency and focus on the moment and the focus is less narrow and more global in time and space. Behavior is guided in a feed-forward manner that can plan for the future, consider alternatives, and simulate and predict future events that build on previous experience. In addition, a successful predictive model will tend to be positively biased, emotionally less intense, able to confront negative and positive affective events and, thus, lead to effective coping.

These investigators demonstrate that the two biobehavioral programs are supported by corticolimbic and endocrine functions, forming the ventral corticolimbic control pathways for reactive control and the predictive program comprised of the dorsal corticolimbic pathways. These two cortical systems interact and collaborate in the control of behavior in many situations and actions. The investigators identify clinically relevant conditions under which one or the other model can become dominant, namely conditions due to temperament, unpredictable dangerous situations, experience of trauma, or developmental effects of inconsistent parenting.

The proposed biobehavioral programs are particularly relevant to the understanding and treatment of emotional disorders, such as anxiety and PTSD. Tops and his colleagues propose that treatment approaches could implement a shift from ventral to dorsal streams in individuals with ventral control biases, such as through mindfulness training [60]. Theirs is not a simple dichotomous model but an evolutionary grounded biological system that functions along a broad continuum where responses are reactive in processing novelty and biological salience in unpredictable environments, such as threat, or are predictive in stable environments where the predictive control system ‘runs simulations to predict future events’ [60] (pp. 376–377). These investigators also support this arrangement with large-scale brain networks of the default mode and salience network.

We see our GRIT intervention as a potential approach that can foster such a shift through its training of goal-directed action and its experience-dependent simulation, which is described in Section 2.2 Goal-Directed Resilience in Training (GRIT): A Model. The goal-directed resilience training model proposed here may serve as a clinical implementation and test of PARCS, a collaboration already underway in our commentary [61] on Kalisch and colleagues [62].

#### 1.1.6. Clinically Relevant Concepts

The abundant research on goal-directed action *versus* stimulus-based responding in diverse research areas has no parallel in clinical psychology or psychiatry, where this subject is merely hinted at in such themes as hedonic and eudaimonic well-being or the motivational constructs relevant to the pursuit of goals, such as efficacy and locus of control. Two of these areas are briefly reviewed.

##### 1.1.6.1. Well-Being: Eudaimonia *versus* Hedonia

Hedonic and eudaimonic processes contain elements of stimulus based *versus* intentional goal-directed responding, as inferred from selected writings of the ancient Greek philosophers. Aristippus of Cyrene of the 4th century BCE advocated hedonism as the sole purpose of a good life [63] (pp. 121–122). Momentary pleasure, preferably of a physical kind, is the only good for humans. However, Aristotle rejected hedonism as unworthy, an aim “fit for grazing animals” [64] (p. 7). Instead of tissue experience of happiness or rewards, Aristotle recommends *eudaimonia*, meaning, “virtue”, “excellence”, and “the best within us” [64] through activities that enact a person’s capabilities to a level of excellence and in accordance with reason. By contrast, in their most radical positions hedonists recommend that pleasure be immediately attained by whatever means and regardless of consequence [65] (pp. 230–231). The radical statements of immediate sensory pleasure without delay and consideration resemble the stimulus-based responses we have in mind. Aristotle emphasized means, the process of virtue, excellence, and the development of the best qualities within us that were achieved through action and excellence; the call to cultivate action and excellence resembles the goal-directed responses of our model.

Vittersø has pitted hedonic well-being against eudaimonic well-being. In a paper whimsically entitled “Was Hercules Happy?” Vittersø and colleagues [18] summarize their functional model of well-being. Hedonic feelings are created in states of equilibrium and assimilation when needs are met and goals have been fulfilled, and when contexts are familiar or simple. Eudaimonic well-being reflects feelings such as interest and engagement, emotions that prompt growth behavior, motivate behavior in challenging environments, and represent an opportunity to build skills and develop potentials. These are associated with episodes of disequilibrium and challenge. Although the understanding of hedonic well-being of the Vittersø group resembles a vegetative state of relaxation and not the hedonist immediate pleasure, their sense of eudaimonic well-being comes close to goal-directed action of this paper, namely of engagement, interest, and curiosity.

The studies of Vittersø and colleagues [66,67] confirmed that happiness scores were higher in easy episodes while interest was higher in difficult episodes. Happiness was higher before work on a Sudoku puzzle than during the work. However, interest was higher during work on the puzzle. Curiosity predicted happiness during a task but not after the task was completed. These investigators concluded that *for growth and development to occur, feelings of interest and engagement were needed*; cultivating such feelings would be required in order for life to adapt to opportunities and demands.

##### 1.1.6.2. Goal-Relevant Motivational Constructs and PTSD

One would expect the distinction of goal-directed action and stimulus-driven responding to have emerged particularly in the study of PTSD, the signature disorder to arise from experiences of extreme situations. However, the study of goal-directed action is singularly absent in the PTSD literature. A notable exception is the work by Simmen-Janevska and colleagues [68], who investigated how traumatic stress changes the motivation to achieve goals. Through an extensive review of the literature relevant to PTSD and goal-directed behavior, they found that goal-directed behavior is extensively studied in areas “outside of abnormal or clinical psychology” [68] (p. 2), but that in order to find any association with motivation to achieve goals within abnormal or clinical psychology literature they must review the negative effects of PTSD on goal-relevant motivational concepts, particularly self-efficacy and locus of control. The investigators adhered to the original definitions of self-efficacy and locus of control. Self-efficacy reflects the belief in the ability to succeed and be efficacious and that effort will pay off [20]. Self-efficacy may thus be construed as a measure of the belief in being effective in attaining goals. The locus of control construct [19] captures internal control or the belief that the person’s own behavior controls the events that affect his/her life. External locus of control implies that chance, luck, or fate control the outcomes of people’s lives. Goals can, thus, be attained through one’s own efforts or may be the result of arbitrary luck and forces outside of oneself.

In reviewing the course of PTSD symptomatology over the life span, Simmen-Janevska and colleagues [68] found that self-efficacy, locus of control, self-esteem, and impulsivity/self-control predicted symptom severity. Eight longitudinal studies reported findings that individuals with high levels of self-efficacy experienced fewer traumatic symptoms and, conversely, individuals with low self-efficacy reported more traumatic symptoms. Cross-sectional studies showed that self-efficacy was the most powerful component involved in the development of traumatic symptoms. These findings suggest that PTSD tends to disturb goal-relevant motivations and likely impact goal-directed behavior.

#### 1.1.7. Beyond Dualities

The models reviewed above have not remained stationary dichotomies. In each case, unanticipated direction emerged that charted divergent directions. In every model, mechanisms that support flexibility are uncovered. Thus, motivation is rendered flexible through expressions of approach-avoidance that are not rigid but complex and flexible, or such mechanisms as stimulus evaluation that can range from primitive reflexive response to complex cognitive evaluation [34]. Positive and negative affect typically conceived as dichotomous bipolarities turn out to be independent affective systems that can co-occur and often do but can become opposing poles in challenging situations [44]. Thus, experience of challenge and threat may create bipolarities where they did not exist in calmer situations. Corbetta and Schulman’s [17] early concepts of goal-directed and stimulus-driven attention evolved into the reorienting system designed to flexibly change an organism’s course of action in response to threat or advantage in its immediate environment [55]. Tops and colleagues’ predictive and reactive control of behavior (PARCS) becomes a much broader flexible calibration of control that is sensitively aligned with the extent of predictability of the environment [22,23]. In the well-being literature, discussions of the polarity of hedonia and eudaimonia increasingly acknowledge that they are different processes that are not necessarily bipolar but may co-occur, subsume each other, be irrelevant or an obstacle to each other (for example: [66,67]. Bipolar models have been remarkably productive in generating work that has extended well beyond the original dichotomies.

#### 1.1.8. Summary: Engagement and Withdrawal

The above dual system models describe aspects of what may be termed approach/intentional goal-directed *versus* stimulus environment-based neurobehavioral dual processes that may, to varying degrees, oppose or function independently of one another, though in overlapping ways and with gradations of conscious or unconscious awareness. These dual processes are widely represented at physiological, endocrine, cortical, behavioral, cognitive, and affective levels. Their flexibility and independence have been widely investigated. The above review illustrates models ranging from motivational processes to behavioral inhibition and activation, ideomotor intention-based and stimulus-based action, goal-directed *versus* stimulus driven attention, biobehavioral predictive and reactive control systems, to related themes embedded in hedonic and self-development literature, and self-efficacy and PTSD.

The literature we have reviewed and critically evaluated has helped shape this model by providing a comparison platform from which to view the GRIT intervention and to argue what is added when goal-directed resilient action enters a traumatic scene. Goal-directed action is independent of the environment, a very useful feature underscored by ideomotor theory (e.g., [50]). Engagement and interest are main features of eudaimonia and contribute to personal growth (e.g., [18]). Goal-directed action support agency and prospective action, in contrast to the person as reactive to threat (e.g., [21]). These qualities of goal-directed resilient action will be covered in greater detail in subsequent sections.

### 1.2. Goal-Directed and Stimulus-Based Responding in Clinical Practice

Although the distinction of goal-directed and stimulus-based responding is largely not represented in clinical research, new developments are emerging in the most recent “New Wave” therapies that have transitioned from exclusively treating symptoms to restoring what is missing, including the use of mindfulness approaches, activation, and Acceptance and Commitment Therapy.

#### 1.2.1. Not all Extreme Experiences Result in Trauma

New ways of thinking about PTSD and depression are evident in the increased recognition that not all experiences of extreme situations result in trauma or reactive depression. The more common response is one of resilience [69]. Shifting from a survival to a resilience expectation in the wake of adversity has been an evolution for both science and society. Taking the United States as an illustrative example, there were marked differences in institutional and public responses to crises occurring roughly a decade apart. The responses to the September 11th crisis involving the World Trade Center highlight the human capacity for resilience. Following the Twin Towers attacks, agencies and institutions sprang into action across the United States to offer emergency mental health services. Surprisingly, there was not an epidemic of trauma, but there was a great deal of resilience. Recognizing this capacity for resilience, Levin [70] later urged the public and mental health community to alter their views of resilience: “Resilience rather than pathology should become the standard expectation in the aftermath of trauma.” The importance of the study of resilience was highlighted by the International Society for Traumatic Stress Studies, which dedicated its annual meeting in 2006 to the theme “The Psychobiology of Trauma and Resilience Across the Lifespan” and the 2014 annual meeting to “Resilience After Trauma: From Surviving to Thriving” in the wake of the Boston bombing. This adjustment in focus appears to have had a significant impact on the public. Following the Boston bombing [71], “Boston Strong” became a popular catchphrase that grew up from the citizens and was publicized by media, a response reflecting an expectation of strength rather than trauma, a marked contrast to expectations for the 9–11 response a decade earlier.

#### 1.2.2. New Wave Therapies

Changes in therapeutic approaches that emphasize capacities slightly antedate or overlap with the 9–11 World Trade Center attack and are characterized by Hayes [72] as “New Wave” therapies. For the First Wave of behavior therapy and the Second Wave of cognitive therapy, the reduction of symptoms was the central goal of treatment. The therapeutic focus was on changing maladaptive ways of thinking and feeling to promote improvement in functioning. New Wave therapies, following on the heels of the First and Second Waves, focused instead on restoring capacities as the central goal of treatment, while including elements of traditional therapies and developing more holistic approaches to emotional treatment.

In developing Dialectical Behavior Therapy (DBT), Linehan [73] recognized the need for a modified form of cognitive-behavioral therapy for emotion regulation, and incorporated Zen Buddhist meditation practices that encouraged acceptance, mindful awareness and tolerance of symptoms. Cloitre and her colleagues [74] proposed to bolster stunted regulation and social skills through the Skills Training for Affective and Interpersonal Regulation (STAIR) plus Narrative Story Telling (NST) for mastery of day-to-day emotions and interpersonal problems. STAIR sessions are designed to teach emotional regulation skills, social skills development, positive self-definition exercises, and goal setting and achievement. NST sessions are individual sessions focused on processing the trauma in context of developing both positive life narratives and motivating future plans. Though the program does not overtly seek to stimulate goal-directed action as a critical part of the healing process, we would suggest that the intervention’s moderate success in reducing PTSD symptoms may be due to the motivational NST component.

Najavits [75] identified a gap in the treatment of dual diagnoses, introducing Seeking Safety and adapting cognitive behavior therapy (CBT) for this population. Exploration of past trauma was not part of the Seeking Safety program; new skills rather than symptoms were the focus of treatment. Behavioral Activation is an approach first developed by Lewinsohn [76], and is one that has reappeared in the work of Jacobson *et al.* [77] and, most recently, of Dichter *et al.* [78]. Well-Being Therapy by Fava and colleagues [79] treats residual symptoms of mood and anxiety disorders, following successful primary treatment of these conditions.

These interventions share the belief expressed by Ryff and Singer [80] that the absence of illness is not equivalent to health and well-being. Recovery should not only alleviate negative symptoms but also foster positive wellness; therapy should not necessarily reduce symptoms but increase personal effectiveness. Perhaps the most innovative approaches belong to those therapies exploring the inclusion of mindfulness meditation in established therapies. These approaches do not necessarily solve problems. Rather, broadly speaking, thoughts are to be observed by the client without judgment in order to increase tolerance of difficult emotions. Examples of this approach are Acceptance and Commitment Therapy (ACT) [72], and Mindfulness-Based Cognitive Therapy (MBCT) [81].

It is interesting to note that this increasing de-emphasis of symptoms as the primary or only treatment focus is occurring at a time when the *Diagnostic and Statistical Manual* has undergone its fifth revision (DSM-5). To be sure, the definition of PTSD and its symptoms are clarified, reinforcing the dominance of classification and a symptom-based approach to mental health with little room for actual processes that produce and maintain mental health. Thus, criterion A includes actual or threatened events: exposure to sexual violation, death, or serious injury. The event must be witnessed or experienced directly, but may be something the affected person has learned of in the case of violent or accidental death of a family member or close friend. Criterion B requires at least one intrusion symptom. Criterion C requires at least one avoidance symptoms. Criterion D requires two negative alterations of cognitions and mood, and Criterion E requires two alterations in arousal and reactivity [82]. Though dissociative symptoms such as flashbacks and psychogenic amnesia are included are general diagnostic criteria, DSM-5 also recognizes a dissociative subtype based on empirical evidence that a subgroup of PTSD patients experience additional symptoms such as derealization and depersonalization [82]. The importance of this subgroup to our work, and that of other PTSD researchers, is the need to recognize that PTSD patients may be experiencing different states of arousal and that trauma-focused therapy approaches require that clinicians attend to signs of fright, flight, or physiological arousal states during the intervention [83].

But are the New Wave therapies or, for that matter, the older therapies not also goal-directed? Do the therapies not have goals, such as correcting cognitions or re-experience of trauma? Do the patients not have goals, such as overcoming their symptoms of anxiety and nightmares? Indeed, both therapists and patients have such goals and others. However, having goals for therapy is not the same as having a capacity to formulate goals, carry them out, approach life with interest and curiosity, imagine a future and formulate life goals. Prominent symptoms of PTSD demonstrate goal impairment in avoidance, a foreshortened future, diminished positive emotions that could foster approach and social relatedness. It is this diminished capacity for goal-directed action and relatedness that this review addresses. We turn next to specific areas of resilience research that contribute conceptually to the GRIT intervention framework.

### 1.3. Resilience

As a field of research, resilience has evolved considerably over the decades from the narrower view that resilience may be measured by a return to homeostasis or sustainability of prior activities to its presently robust model of positive adaptation to adversity, which suggests an additional process of personal growth or meaning making [8,13]. As with other fields, methods have tightened up with better operationalization and measurement of resilience constructs, with clearer explication as to whether models consider resilience as process or as outcome, or constructs that represent both resilience-related processes and constructs. One resilience process that relates closely to our model is cognitive shift, a dynamic process of coping in which a person faces an event that produces chronic, unremitting stress requiring exceptional adaptation and discovers new goals, behaviors, or ways of thinking that support positive affect and personal resources; cognitive shift is unique because it recognizes the imperfection of transformative processes, *i.e.*, that coping with complex stressors involves some period of oscillation between past and future coping orientations before a primary shift towards positive adaptation takes place [12]. We describe a positive adaptation that connotes personal growth emerging out of goal-directedness and context independence. Despite the prolific and often imprecise use the term ‘resilience’ in the literature [7,84] we will specifically use ‘goal-directed resilience’ and ‘goal-directed skills’ to describe the adaptive actions supporting growth.

#### 1.3.1. Resilience Training as Primary Prevention

Resilience training has emerged in the form of primary intervention programs to prevent maladaptive development in children at risk due to parental mental illness, incarceration, poverty and other adverse conditions. Resilience interventions seek to provide support against risk factors and/or develop competencies believed to promote long-term health and measure outcomes that could indicate the success of these aspects of the program. Prevention programs are typically developed and evaluated on two main dimensions: (1) whether the intervention is centered on the person or the environment; and (2) selection of target samples via approaching the entire population, an at-risk group, or a group that has experienced a life event [85]. We will review work with two populations where primary intervention programs have flourished: developmental resilience interventions and resilience training for the Armed Forces. Our own model of goal-directed resilience training has focused on secondary intervention and will be reviewed subsequently.

##### 1.3.1.1. Resilience Prevention Programs with Children

Developmental prevention programs have range in their level of involvement in participants and their families’ lives. For example, the Houston Parent-Child Development Center Project [86] conducted regular home visits with at-risk families that involved a 2-year, 500 h commitment of the families’ time. Outcomes indicated improvements in child-rearing behaviors, interactional styles, and children’s enhanced cognitive development and adjustment.

Other developmental programs feature greater brevity, targeting a specific risk event such as divorce and providing children at risk for traumatic fallout from the cascading secondary stressors set in motion by divorce with education and coping skills. An example is the 11-session New Beginnings program [87], which focuses on coping, negative thought reduction, and improving the parent and child relationship. Some long-term benefits of the program for the child participants include low externalizing symptoms, fewer symptoms of mental disorders, and less drug and alcohol use than children not participating in the program.

##### 1.3.1.2. Resilience Prevention in the Armed Forces

The Armed Forces have been challenged to balance the warrior culture with the need to prevent the development of anxiety and PTSD symptoms. For the military culture, prevention strategies require training, emphasis on unit cohesion, and leadership [88]. Considerable resources have been allocated by the US Department of Defense to promote resilience among soldiers, families, and those providing care to soldiers [89]. The US Army approaches resilience and prevention of post-deployment traumatic symptoms with its Comprehensive Soldier Fitness and Battlemind programs [88]. The Comprehensive Soldier Fitness Program consists of four primary components, or pillars: psychological fitness assessments, resilience training, individual training, and master resilience training [90]. The Master Resilience Training component is the only pillar that has been measured against program goals and well-published. The purpose of the component is to teach non-commissioned officers resilience skills, such as self-awareness and self-regulation, over a 10-day period with instruction on how to teach these skills to the soldiers in their units. This component demonstrates small effects in improving both emotional and social fitness outcomes when comparing units without trainers [90].

Battlemind was mandated for soldiers post-deployment in 2007 but has now been scaled to include soldiers, families, leaders, and at-risk groups at various stages in of pre- and post-deployment. Battlemind is considered a stress management program, teaching participants that Battlemind is an acronym in which each letter stands for skills soldiers develop that may be useful on the battlefield but must be readapted after returning home to avoid causing problems. Examples are: “Buddies (cohesion) *vs.* withdrawal”, “Targeted aggression *vs.* Inappropriate aggression”, and “Tactical awareness *vs.* Hypervigilence”. The program combines active engagement with one’s internal and external environment with the support system provided by the military culture, making it one of the military’s longer-running mental health programs. Success of the program is not fully established, based on the limited available evidence. However, it appears that in a study of 1060 soldiers completing a follow up assessment at 4 months, the Battlemind Debriefing program may be associated with small improvement effects on sleep, depression, and PTSD outcomes [91].

#### 1.3.2. Resilience Training as Secondary Intervention

While resilience training for prevention has remained modest, resilience intervention has exploded in a burgeoning of programs and publications. This is evident in the number of major edited volumes published during the past five years alone e.g., [92,93,94,95,96,97], including two handbooks by collaborators of this intervention program [2,98]. Resilience behaviors have many expressions: music reduces stress, so do positive emotions, touch, social relationships, empathy, volunteering, and many others. Resilience occurs in many contexts: within the mind, in interpersonal interactions, small groups, the community, and others. A larger review is beyond the scope of this paper. For larger overviews, the reader is referred to the above references. Instead, we offer our research program as an example of the development and testing of a specific intervention model.

#### 1.3.3. Resilience Training as Restoring and Training Goal-Directed Skills: A Model

The model described here is a resilience intervention that restores and trains compromised goal-directed skills in evocative contexts. Comparable to the New Wave approaches, we set out to train missing skills. These were skills we came to identify from people’s narratives of *exceptional* survival in extreme situations, with a particular focus on how their actions promoted adaptive survival. We therefore conducted a qualitative narrative inquiry see [83] into a broad literature comprised of classical survival narratives (*n* = 16) for behaviors that enabled people to persevere under extreme personal challenge. The single concept that guided this search was “adaptive survival” or comparable language used by survivors themselves, by knowledgeable observers, or experts. Going back and forth between text-based, video, and photographic evidence of survival in response to severe circumstances, we identified themes in the data that informed the design of this intervention.

Two prominent themes of adaptive survival emerged: approach/engagement and social relatedness. *Approach/engagement* was expressed in acts of interest, curiosity, appreciation, and noticing beauty. *Social relatedness* appeared in examples of empathy, compassion, helping, friendship, and love. These two themes exhibited a number of characteristics:

(1) Goal-directedness: The actions in the examples of engagement and the social relatedness examples were *goal-directed*. As reviewed earlier, the ideomotor theory defined goals as rooted in responses made to the effects or consequences of actions. Poetry chanting by Eugenia Ginzburg, a Russian journalist who became a political prisoner for 18 years in the Gulag, exemplifies this well. One line of poetry anticipates and has as its goal the chanting of the next line. Parenthetically, it can be noted that skilled performance is goal-directed.
(2) Emotional valence: The actions of survivors were ones they valued and were accompanied by a certain *emotional intensity*, even passion.
(3) Context independence: These actions were *independent* of the threatening context. This independence came as a result of the actors responding to the consequences of their actions rather than to the threatening environmental stimuli. This feature is easily missed and is so central that it requires to be underscored as a particularly adaptive element.
(4) Altered environment: These goal-directed actions changed the *environment* or experiences of the environment whereas merely reacting to threat, or reactivity, changed the person.
(5) Engagement/Social relatedness: Finally, the actions of *engagement* as expressed in interest, curiosity, or appreciation and *social relatedness* of empathy, compassion, or helping become resilience skills and life-preserving abilities when expressed in adverse contexts. They become the focal activity that holds its ground and prevails in the face of adversity.

Our qualitative findings are consistent with findings from the developmental literature on children growing up in adversity. The positive outcomes of these children are often associated with having a close relationship with one or more adults and with being effective in the main areas of their lives e.g., [99,100]. Our findings on adult exceptional survival add more clearly the specific characteristics of adaptive survival: actions that are *goal-directed*, are *emotionally grounded*, are *independent* of the threatening context, change the environment, and have adaptive effects and, thus, represent resilience. These qualities of goal-directed adaptive survival encompass more complex and nuanced aspects of responding than is associated with the notion of resilience as a return to homeostasis or sustainability of prior activities. The model of resilience training we describe here promotes personal growth emerging from goal-directedness and context independence.

#### 1.3.4. Iconic Exemplars of Resilient Action

The narratives we analyzed covered episodes of human inflicted violence from events of the past century, namely the Gulag, the Holocaust, Vietnam War, bombing of a city, and a kidnapping in the United States. The format in which narratives appeared were varied; some were captured on video e.g., [101] while others were autobiographical accounts e.g., [102]. For our research purposes, the construct of interest was a product of the retrospective meaning made by the perceiver. As long as the method of recording the narrative captured the perceiver’s reported account of adaptive survival, our decision was not to exclude any medium for recording such an account. Below we review several case studies that served as iconic examples to which we repeatedly returned in our efforts to understand adaptive survival in clear and nuanced ways.

##### 1.3.4.1. Approach/Engagement

**Interest and curiosity**. Robert Shumaker became a prisoner of war (POW) in 1965 when he was held in the notorious ‘Hanoi Hilton’ prison in North Vietnam. In a televised documentary [101], Shumaker described building his dream house in his imagination and counting the number of nails and feet of lumber he would need. He became curious about changing the design, moving a fireplace, and modifying his design. To communicate, he and fellow inmates devised a tap code which they also used to learn and teach each other various subjects. When asked, he affirmed that he would not wish away his captivity: “I had learned things about myself. I learned tools, psychological tools, tools in ways to handle my life that I probably could not have learned in any other way. So it stood me in good stead for the rest of my life” [101]. He concluded that, if he could survive captivity, he could survive anything.

**Appreciation and noticing beauty**. 1937 is known as the year of the Great Purge, of sweeping purges and the imprisonment of millions in the Siberian prison camps or Gulag of the former Soviet Union. Eugenia Ginzburg spent 18 years in the Gulag, the first year and a half in solitary confinement. She chanted poetry in her solitary cell, poetry she had learned in earlier years. As she chanted, she could sense Pushkin and other poets in her cell. She thought that with poetry she could survive any dungeon [102]. Indeed, she wrote her own poems and her mind shaped the memoir she would write later: “Just remembering it all to record it later had been the main object of my life throughout those eighteen years” [103]. Marcella Leet heard of a similar episode that was described to her by a patient. As a young boy during World War II, the patient played his violin whenever his city was bombed. He heard bombs, saw smoke rising from burning areas of the city, yet he followed the notes of his musical piece [104].

##### 1.3.4.2. Social Relatedness

**Helping others.** A group of children were kidnapped in 1976 in Chowchilla, California. They were transferred from their bus to two vans, taken to an underground vault or cave and left there. The roof of the vault started to collapse and the children feared they would perish. One of the boys dug a way to the outside and helped his classmates to safety. Several months later, the psychiatrist Lenore Terr [105] interviewed the children. She found that all of the children showed signs of stress except for the boy who had found a way out and had helped others escape.

**Valuing love over hate**. As a medic, George Ritchie [106] was assigned to an American unit that liberated a Nazi concentration camp near Wuppertal at the end of World War II. In attending to survivors, the Americans encountered a survivor who spoke several languages and was very helpful. He appeared fairly fit and in good spirits. They nicknamed him ‘Wild Bill Cody’ because of his handlebar moustache. They thought he looked so well probably because he had spent only a few weeks in the camp. In fact, this survivor had seen his wife and five children executed before he was imprisoned himself in 1939. As a former attorney, he had seen what hatred had done to people. He resolved not to hate anyone but love everyone and be helpful. He-had spent six years in captivity. Although he had had the same rations and treatment as everyone else, he had survived so much better with compassion and caring: ‘I had to decide right then whether to let myself hate the soldiers who had done this. It was an easy decision, really… Hate had just killed the six people who mattered most to me in the world. I decided then that I would spend the rest of my life—whether it was a few days or many years—loving every person I came in contact with” [106] (p. 116).

**Empathy and Compassion**. In the midst of brutal circumstances, there are amazing examples of empathy and compassion. Primo Levi was a young chemist in Italy when he was arrested and set to Auschwitz in February 1944. Short and slight of stature, he was usually assigned tall bed bunk partners, a disaster because he would not get good sleep. He reports on one exception: “But it could at once be seen that Resnyk, despite everything, was not a bad companion. He spoke little and courteously, he was clean, he did not snore did not get up more than two or three times a night and always with great delicacy” [107] (p. 58). Empathy and compassion can be shown through quite minor considerations: it is notable that Resnyk was able to show such consideration and Levi was able to notice it.

Another example comes from Charlotte Delbo, a member of the French Resistance who was captured and sent to Auschwitz in 1943. She describes how the prisoners in her group kept each other warm while waiting for roll call: “Neck drawn into her shoulders, chest pulled in, each places her hands under the arms of the one in front of her. Since they cannot do it in the first row, we rotate. Backs to chests, we stand pressed against each other, yet, as we establish a single circulatory system, we remain frozen through and through” [108] (p. 63). And thus they tried to prevail with such small, hardly visible acts of kindness.

#### 1.3.5. Simulation of Goal-Directed Resilience

We set out to consider ways to simulate the above adaptive resilience responses with groups whose symptoms were entrenched reactions to threat, such as PTSD. Large areas of diverse basic research addressed the centrality of action, simulation of action, simulation of intention, anticipation of action in neuroscience, linguistics, artificial intelligence and others. Central cortical mechanism supported the making and manipulation of internal models of action. Such manipulation allowed behavior to be freed from a present-orientation and stimulus-based responding and to be become prospective and future-oriented (e.g., future goals that direct actions rather than reactions induced by the environmental).

These studies showed that the making of inner models and the simulation of anticipation made possible disengagement from present motor activities so that new goals and alternative actions could be formulated. Taking an example of internal model-making as coping from the narratives, while imprisoned in the Hanoi Hilton, Shumaker created an internal model of his dream house, calculated the materials he needed, and changed elements in it. Internal models are accompanied by sensory experiences that occur in the moment they are being created. Well established findings showed that diverse imagined activities produced a wide range of sensory inputs that activated cortical areas in ways similar to those also activated by actual action: imagined action [109,110], sounds associated with action [111], language of action words [112], and touch [113]. All these diverse sensory inputs from various imagined acts activated cortical motor areas that were also activated by actual activities and were accompanied by similar sensations. For example, to imagine a walk and taking a walk for a certain distance took a similar time. The heart rates of people engaging in imagined and actual activities are similar [109].

These findings suggest that simulation can be powerfully incorporated into training approaches. These basic neuroscience findings on simulation and anticipation would bolster an experience-dependent simulation aimed at transforming reactivity into anticipatory action. Simulation and sensation are central components of goal-directed resilience training. Since sensations are key effects of simulation, the focus on sensation is also a key element of training in our model. Moreover, it is at the level of sensation that experience first enters a person’s life and, at this juncture, can become dominated by fear or withdrawal, as in PTSD. At this level of sensation, dominant symptoms could be more deeply transformed into prospective anticipation and goal-directed action in a more bottom-up approach [109].

#### 1.3.6. Summary of Survivor Themes

We qualitatively analyzed narratives of people who faced life-threatening circumstances with two types of responses: *approach and engagement* with their surroundings in ways that promoted wellbeing, and *social relatedness* in which they sought out and expressed human connections. In the midst of unpredictable threats, narrators described actions and relatedness that were: goal-directed, emotionally grounded, context independent, and changed the environment or changed the experience of environment.

## 2. Method

The Development of a Goal-Directed Resilience Training Model.

### 2.1. Background

We developed the GRIT intervention aimed at restoring goal-directed adaptive functions. Our approach took as its model natural resilience expressed in extreme situations. The aim of the intervention was to bring the qualities seen in exceptional survival into participants’ personal experiences of trauma and thereby simulate a resilient response that sufferers from PTSD did not previously have. Thus, participants would respond with goal-directed actions resembling those of exceptional survival, with resilient action rather than entrenched trauma. The aim was twofold: 

#### 2.1.1. Simulation

**Figure 1:**
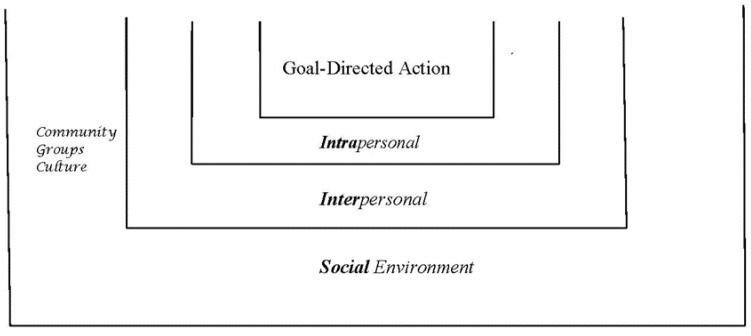
Three contexts of obstacles within which people practice their goal-directed adaptive actions. The obstacle, trauma, challenge become the ‘stage’ or backdrop for goal-directed action that is played out on stage. Experiences of engagement as in interest, curiosity, appreciation or of social relatedness as in empathy, compassion, helping are enacted on these stages where obstacles are the backdrop or scenery and not the main actors. The stages can be: internal or ***Intrapersonal*** and represent states of mind and emotions or interoceptive body states such as pain; or ***Interpersonal*** such as conflict with another person; or ***Social*** as exemplified by experiences with larger groups, the community, or culture.

Through simulation activities, we sought to simulate adaptive responding (see Figure 1. We have dubbed the adaptive survival themes from the qualitative analysis as ‘life skills’ for the intervention. These skills are expected to be useful in challenging contexts because of: (a) their prospective, goal-directed qualities; (b) their independence from threatening context; and (c) the positive, affirming emotions accompanying them. Evidence suggests that these life skills activate prospective goal-directed cortical mechanisms and executive functions, while supporting deactivation of stress functions at cortical, endocrine, and physiological levels [14,114]. The effect is prospective goal-directed action and affective engagement that has an ameliorative effect on stress. Supporting work for disengagement of PTSD symptoms from executive functions is found in the work of Aupperle and colleagues [115]. Thus, we posit that these life skills are likely to be most helpful in environments or contexts that tend to strongly evoke emotional and physiological reactivity, such as: severe physical or social threat, interoceptive bodily contexts such as chronic pain, and chronic physical illnesses with a significant disease burden.

#### 2.1.2. Grounding New Skills in Sensation

We have adopted a method of simulation and repeated practice that addresses multiple levels of functioning simultaneously: physiological, cortical, endocrine, cognitive, affective, and motivational. In this approach, participants identify earlier experiences of engagement and social relatedness, describe and re-experience them by describing in detail the reactions of their five senses. To expand re-experience even more, participants are asked to make visual representation of them. Participants are next asked to take these experiences into episodes and contexts of old threat or trauma. This process was believed to modify the re-experience of threat itself at a deep, neurobiological level and to induce interoceptive bodily homeostatic changes. The neural pathways involved in such embodied covert simulation, implicit anticipation, and bodily anticipation, are summarized in detail by Svenssen, Morse, and Ziemke [116].

### 2.2. Goal-Directed Resilience in Training (GRIT): A Model

We next describe the main method we have developed for GRIT, its basic components, and its implementation in clinical trials. We will conclude with the development of a model for testing the effectiveness of resilience skills training in PTSD and discuss its broader adaptation to evocative contexts that include obesity and chronic pain.

GRIT, or Goal-Directed Resilience Skills Training, is a structured, manualized program that takes participants through four steps in which they re-experience past engagement and relatedness episodes and use these in simulations to transform trauma:

(1) **Preparation**. To allow participants to do the work of the program, a readily available calming response is required. Participants are asked to set traumatic experiences aside and find an episode from childhood or early adult years in which they are cherished and loved, or they cherish and love someone or something else. When stressed during the intervention, they are to return to this episode rather than remain stressed. Experiences of secure attachment restore feelings of safety even during high threat conditions and aid in healing trauma even when mobilized symbolically [117].
(2) **The elements**. Participants identify approach/engagement and social relatedness experiences from childhood and early adulthood. Since these experiences are not novel but are already biologically established, they facilitate the re-experiencing of goal-directed responding and ground these in sensations. The view of memory as a construction made up of fragments of the past [118] is particularly compatible with the ‘simulated constructive’ approach of the program. Participants have an opportunity to find relevant past fragments and fashion new themes out of them.
(3) **Transformation**. Approach/engagement and social relatedness episodes are used in a return to traumatic events. As noted earlier, engagement activities of interest or curiosity and social activities in themselves are relatively pleasant and innocuous. However, they become tools for transformation when facing challenges, thereby demonstrating adaptive action and ‘resilience’. A case example of transformation from past trials of the intervention comes from a Vietnam-era veteran who returned to a battle scene in his narrative with the activity and sensations of holding his first frog in his hands when he was five years old. The battle scene felt like the wiggling magical creature in his hands. Another case: a veteran returned to Baghdad as the boy fixing his toys and selling them on the sidewalk in Cleveland. The veteran described the sensations of the Cleveland sidewalk sale in Baghdad, the humid air of Ohio in Baghdad. The bombing in the Baghdad market took on a different feel and perspective. In this constructive approach to memory and sensation, the past is rearranged into a recombined memory with goal-directed action that is grounded in sensation and a prospective direction to the future.
(4) **The future**. The goal-directed approach/engagement and social relatedness are applied to designing a good life with resilient responses to possible future challenges. Participants’ futures contain their own goals and interests and pathways to achieving them. Goal-directed engagement and relatedness are essential for flexible anticipatory adaptation capable of transforming reactivity. This process enables participants to create a new, resilient, and more integrated narrative of their lives that is rooted in their experiences and sensations.

A detailed manual covers the above steps in four modules that are described in Table 1. The manual serves as an outline and guide for facilitators and participants.

**Table 1:** A Modular Program for Resilience Training of Action and Agency.

Introduction	**Introduction and Psychoeducation:** Participants find an example of cherishing. The overview of the program covers the main concepts of goal-directed resilience training: Approach/engagement and social relatedness. The four modules are introduced: (1) identify and re-experience past approach/engagement experiences; (2) identify and re-experience past social relatedness; (3) use past experiences in simulations to transform stress and trauma; (4) build a good life. Participants practice activities that illustrate these concepts.
Module I	**Approach/Engagement:** The content covers interest, curiosity, appreciation, noticing beauty. These are reinstated and expanded by re-experiencing past episodes of childhood and early adulthood, times that are formative. Participants are asked to describe each episode in detail. They are asked to describe the sensations that accompany each episode, drawing from their five senses (vision, hearing, smell, touch, taste). Participants are to make a visual representation of approach/engagement using a medium of their choosing (e.g., collages, sculptures, *etc.*). Brain and endocrine functions are briefly reviewed with neuroscience examples provided. Reading and visual examples are provided, as are activities. Homework is assigned.
Module II	**Social relatedness:** The content covers experiences of empathy, helping, friendship, and love. These are re-instated and expanded by re-experience of past episodes of social relatedness from childhood and early adulthood. Participants are asked to describe each episode in detail. They indicate how they feel in each episode with their five senses: seeing, hearing, touch, smell, and taste. Participants are to make a visual representation of social relatedness. They are to choose the medium with which to represent their personal examples. Brain/endocrine functions are reviewed with neuroscience examples provided. Readings and activities are provided. Homework is assigned.
Module III	**Transformation:** Simulation is used to integrate the newly re-established engagement and relatedness experiences of Modules I and II with an evocative context such as a stressful life event. Challenging experiences are revisited in a graded manner with the above practiced engagement and social relatedness experiences. Participants identify stressful events or challenges they wish to return to. The challenges should be graded as to the degree of distress they cause. Participants choose less challenging ones first and leave more distressing experiences for later. Participants are to return to a challenging event with the resilience skills of engagement or relatedness. Participants are asked to describe this resilience-based return to challenge and describe the sensations that accompany the resolution with their five senses. Challenges come from several problem areas or ‘contexts’ of a person’s life: intrapersonal, interpersonal, groups, the culture. These can be represented visually as a theater stage in which the participant is the actor. Participants are to take approach/engagement or social relatedness examples onto the various stages and discuss their lives in these contexts. Reading examples and activities are provided. Homework is assigned.
Module IV	**Building a Good Life:** Participants will be asked to identify the key characteristics of a life well lived. Participants are asked to imagine and design a life well lived. Describe a good life for themselves. Participants are asked to describe a good life with a future. How are they proactive and engaged? How are they embedded in social relationships? The module will discuss the Greek definition of a good life as “the exercise of vital powers along lines of excellence in a life affording them scope.” Most lives are limited in various ways and do not automatically have “scope”. This module will discuss a good life as one that creates scope in the midst of limitations. Participants are asked to create ‘scope’ in their own lives, using the life on stage exercise covered in Module III. Reading examples and activities are provided.

Past experience, sensation, and simulation thus play important roles in goal-directed resilience training. Current conceptions view episodic memory as a re-constructive system that informs future thinking. According to Schacter and Addis [118], the main function of episodic memory is likely not the recall of past episodes. Rather, episodic recall finds past bits and pieces, and then constructs and simulates possible future scenarios, outcomes, goals and needs. This constructive view of memory is very compatible with our experience-dependent simulation in which pieces of the past are rearranged in order to transform trauma. Rather than focus on the details of an episode, the training emphasizes the sensations carried by the details. Sensory memory of approach/engagement and relatedness help to alter the sensations of past trauma at a deeper sensory level, changing the sensations of a dreaded and foreshortened future to a future participants are engaged in shaping.

The activities in Steps 3 and 4 are described as *experience-dependent simulation*, since the simulations are drawn directly from participants’ personal experiences. Studies on simulation demonstrate the ubiquitous presence of simulation in everyday thought, that they match social events, represent causal action sequences, and seem especially real, valid, and useful in solving problems. People simulate highly specific settings in which behaviors match real actions and social reality [119]. By solving problems and serving emotion regulation, simulations evoke actions and emotions that can counter stress and trauma, thus restoring prospective actions and related affects. The resulting changes in affect, symptoms, and cognition happen concurrently, effecting the transition from reactive stimulus-bound responding to prospective goal-directed action.

Below is an example of a simulation exercise from the manual in which participants are invited to describe their examples of engagement and relatedness and place them on a theater stage. The stage represents harmful contexts from their lives (e.g., the childhood example of selling toys in the threatening context of a bomb explosion in Baghdad). The activity “Life’s a Stage with Three Sceneries” was inspired by Shakespeare’s analogy, “All the world’s a stage, and all the men and women merely players” [120]. Figure 1 illustrates three stages that represent three main types of contexts.

**Figure 2:**
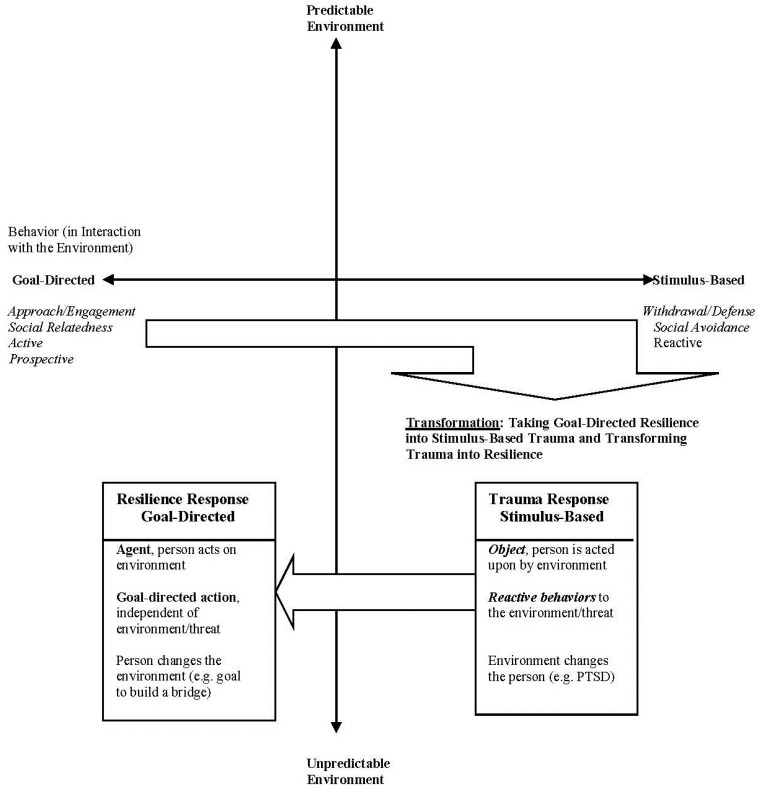
Schematic representation of goal-directed actions transforming stimulus-based responding. These two basic modes of interacting with the environment become visible in extreme situations where adaptive responses are goal-directed actions and trauma is expressed as stimulus based reaction. During resilience training, goal-directed actions are taken into past experiences of trauma, as indicated by the prominent forward arrow leading from the goal-directed pole to stimulus-based pole as experienced in trauma. The transformation is brought about by the qualities of goal-directed action: the person is an actor rather than reactor or object being acted upon by the environment, the action is independent of the environment, and the person changes how he/she experiences the environment or changes the environment itself. In this way, the person no longer is reactive to the environment, is no longer changed by the environment but is an agent, goal-directed in his/her actions, and affects changes related to the environment, as indicated by the reverse arrow.

In the above exercise, participants complete the above stage simulation with examples from their own lives. For example, an explosion in a market in Baghdad is the stage or scenery and selling toys on the sidewalk from a childhood is the action in the Baghdad scenery. Participants are also provided with readings that illustrate challenges and responses in the intrapersonal context, the interpersonal context, and the context of the social environment. For example, Helen Keller is identified as someone responding to the intrapersonal physical context of impairments in hearing, vision, and speech. For her these had severe consequences until she learned the goal-directed skill of signing and communicating. Rosa Parks is someone who faced the external social environment of threats from racial discriminatory practices. Both individuals created opportunities for their development out of and despite the barriers in these different contexts. The transformation of stimulus-based responding to goal-directed action is summarized in Figure 2 below.

It is in Figure 2 where the underlying mechanisms become visible and show how goal-directed action transforms stimulus-based responding. To reiterate, the mechanisms are the qualities of goal-directed action: independence of actions from the environment, actions change the environment while stimulus-based responses change and diminish the person, and the actions are performed by an actor rather than the person being acted upon, an object who is reacting. The traumatic experience becomes background from which the goal-directed action is independent. These features also become more recognizable when viewed in the context of the relevant research literature reviewed earlier. From this vantage point, when we take goal-directed action into a threatening environment, it is no longer applicable to describe the action and the environment in terms of positive or negative valence, or intensity of motivation. Such an approach misses the adaptive goal-directed action. In addition, the concepts as predictive and reactive control are applicable, but the mechanisms of goal-directed action have broader effects, such as how the environment is experienced, changing the environment itself, or how personal growth emerges from pursuing own goals and becoming an agent. Thus, goal-directed action itself points beyond the duality of action and reactivity.

### 2.3. A Model for Goal-Directed Resilience Training in Evocative Contexts

The goal-directed resilience training program was first developed for veterans diagnosed with PTSD. We have recently applied this training to other conditions that share characteristics with PTSD, specifically obesity and chronic pain. PTSD is frequently comorbid with these conditions, which also share physiological, cortical, behavioral, affective and cognitive features resembling those of PTSD. Our approach to identifying similarities and differences across diagnostic categories as targets of investigation is in keeping with the National Institute of Mental Health (NIMH) Research Domain Criteria (RDoC) project [121]. Several common features are summarized in DSM-5 reviews [122,123] and other relevant studies e.g., [124,125,126]. Samples of commonalities are listed below:

**Physiological Features**
  (1) Stress and dysregulation of the hypothalamic-pituitary-adrenal (HPA) axis in PTSD and chronic pain.
  (2) Changes in cortical circuits that include PFC dysregulation and changes in the reward circuitry in all three conditions. Dysregulation of reward circuits.
  (3) Change in energy regulation and sleep.
**Environment as Precipitant**
  (1) Environmental precipitants that include childhood trauma and adult trauma.
  (2) Hyperresponsivity to environmental stimuli, salience of threat or reward stimuli.
**Behavioral Features**
  (1) Interference in social functions.
  (2) Interference in daily activities and work roles.
  (3) Change in reward system such that normal rewards from valued activities and social relationships are no longer rewarding and are replaced by avoidance of threat, seeking cessation of pain, overeating.
**Cognition**
  (1) Attention is automatic and reflexive, with a negativity bias in PTSD and pain, reward salience in obesity, salience of threat and pain in PTSD and chronic pain, hypervigilance in PTSD.
  (2) Dysregulation of executive functions in all three conditions evident in the reduced ability to inhibit responses and pursue goal-directed action in order to realize future goals.
  (3) Learning is rapid and implemented by principles of conditioning, rather than slow learning, as in the learning of skills.
**Chronicity**
  (1) All three conditions are chronic and are difficult to terminate or reverse.
  (2) All three curtail new experiences, new learning, and personal growth.
  (3) The results are a constricted range of experiences and an impoverished life.

In adapting goal-directed resilience training to obesity, we began with processes that maintained overeating, particularly reward salience, conditioning that had evolved into habits and compulsions, and impaired executive functions seen in impaired inhibition and reduced goal-directed future orientation. Our strategy was to expand sensation beyond food to include experiences of the five senses and thus recalibrate sensory experience. The modules of engagement and social relatedness expanded experiences beyond dysregulated behavior, cognition, and affect through the engagement of interest, curiosity, goal-directed behavior, and social functions. The program concluded with designing a life with a broader range of goals and experiences in which food was one interest among others.

GRIT was adapted to chronic pain in a comparable manner that recognized unique characteristics of pain as a warning call to action beyond the relief of pain. A review of mechanisms that help maintain chronic pain for each individual and the recalibration of sensory experiences were also important components. Engagement and social relatedness introduced approach experiences and satisfaction of goal-directed action in the face of pain. A future designed with a broader range of plans and experiences completes the chronic pain resilience skills training program.

### 2.4. A Model for Testing the Efficacy of Goal-Directed Resilience Training

We conclude this overview of the resilience skills training program with a description of a model we have followed for testing the efficacy of our intervention. We conducted our first preliminary feasibility studies in small uncontrolled trials with veterans diagnosed with PTSD [127]. The frequent co-occurring comorbidities of substance use and chronic pain led us to expand the feasibility studies to small group trials of PTSD with comorbid substance use and a third group of PTSD with chronic pain. These early efforts informed the development of the content we have described in this paper. The pilot studies were indispensable in identifying suitable outcome measures, particularly since the intervention aim was to affect a broad range of functions and be applicable to a wide age range in order to represent veterans who had served in the major U.S. conflicts: Korea, Vietnam, and the Persian Gulf. The recent and current wars in Iraq and Afghanistan had barely begun. Inclusion criteria for these early feasibility studies were a CAPS score >40 while exclusion criteria covered active suicidality, psychosis, active alcohol/substance use, and life-threatening illnesses. Group size in each trial was limited to 10 participants who were referred by VA Hospital clinicians.

The efficacy of these trials was assessed with standardized, well validated, and frequently used measures that were chosen to assess four domains: level of symptoms, level of well-being, cognitive functions, and physiological/endocrine/cortical functions. Examples are:

(1) **Symptom measures**: Clinician Administered PTSD Scale (CAPS) [128], the PTSD Check List [129], the Beck Depression Inventory-II (BDI-II) [130], State-Trait Anxiety Inventory (STAI) [131];
(2) **Well-being measures**: Vitality and Social Functioning subscales of the RAND 36-item Health Survey [132], subscales of the Psychological Well-Being Scale [133] that include Purpose in Life, Positive Relations with Others and Personal Growth [134];
(3) **Neuropsychological tests**: Executive functions assessed with the Word Generation subtest of the Neuropsychological Assessment Battery [135], the Category Fluency, Category Switching, and Color-Word Switching subtests of the Delis-Kaplan executive Function System [136], Repeatable Battery for the Assessment of Neuropsychological Status [137] subtests assessed working memory and episodic memory. Alternative versions of all cognitive tests were employed at pre- and post-assessment.
(4) **Physiological/endocrine measures**: We explored the feasibility of assessing cortisol changes with salivary cortisol and evaluated several devices for ease of use and reliability to assess heart rate variability.

## 3. Results Supporting the Model for Goal-Directed Resilience Training

### 3.1. Posttraumatic Stress Disorder (PTSD)

Preliminary support for the GRIT intervention comes from the application of the model to evocative contexts that represent experiences of extreme threat as expressed in PTSD, hyperresponsivity to food as expressed in reward salience in obesity, and of chronic pain. We summarize briefly the findings from a randomized clinical trial (for details see: [114]). The study aim was to test the efficacy of goal-directed resilience training in relieving symptoms, improving emotional health and well-being, and improving executive functions and working memory. The study sample was comprised of 39 veterans diagnosed with PTSD who identified trauma experiences as indexed by the CAPS covering 31% combat, 21% childhood sexual abuse, 18% childhood physical abuse. The duration of the sample’s PTSD symptoms averaged 12 years (range= 1–41 years). The sample characteristics are fully described in the cited study. All participants underwent pre-intervention testing followed by random assignment to goal-directed resilience training (*n =* 20) or a wait-list control (*n =* 19) condition. The intervention was conducted in 90 min sessions over 12 weeks using a support group format limited to ten participants in each group. At the conclusion of the intervention, both the treatment and control groups were assessed a second time.

**Figure 3:**
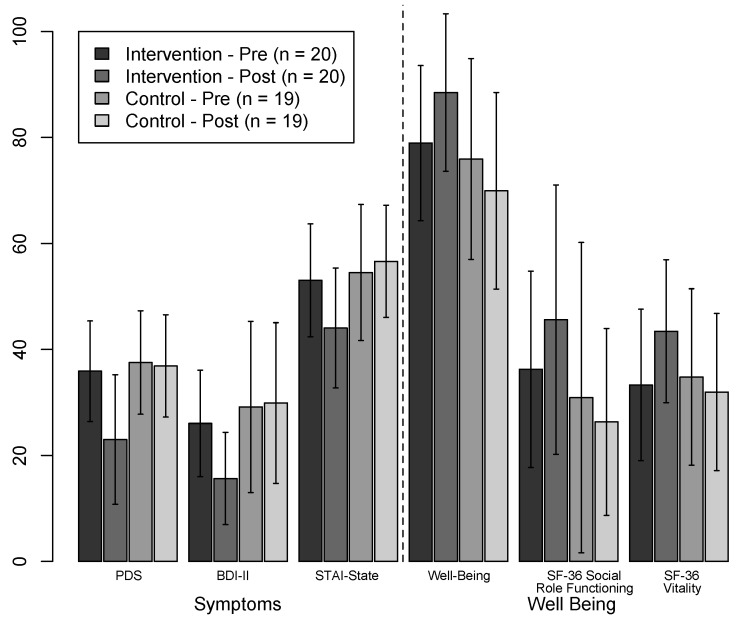
Mean scores on symptoms and well-being for treatment and wait-list control, pre- and post- intervention.

**Figure 4:**
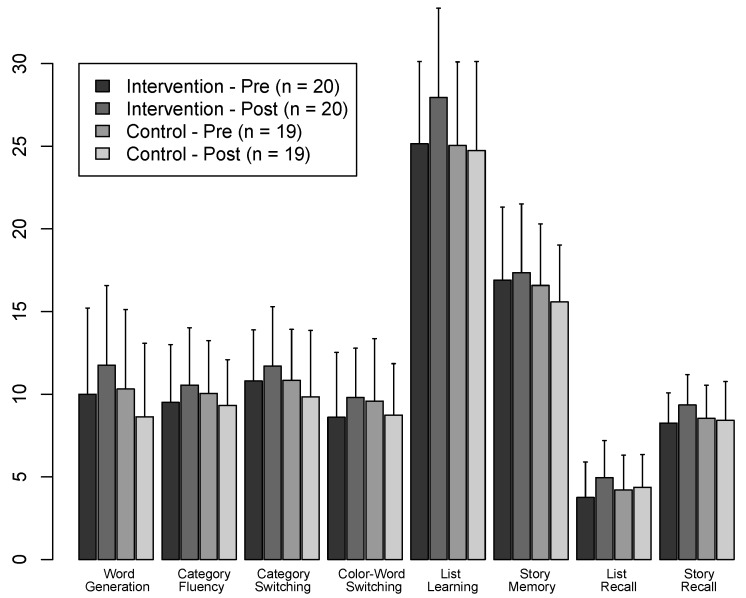
Mean scores on neurocognitive functioning for treatment and wait-list control, pre- and post- intervention.

In descending order of magnitude, the findings showed large to moderate declines for the treatment group in self-reported symptoms, and gains in well-being and neuropsychological test performance. Thus PTSD and depression symptoms declined in comparison to wait list controls, a change that was comparable in magnitude to that obtained with exposure therapy [114]. Indeed, at pre-testing (PDS) 70% of the treatment group scored in the severe range for PTSD. At the close of resilience skills training only 30% of the treatment group scored in the severe range at post-testing. A comparable decrease was obtained for depression in the treatment condition. Substantial pre- to post-test changes were obtained for self-report well-being measures, with gains in Vitality and Social Functioning and the combined Well-Being subscales of Purpose in Life, Positive Relations with Others, and Personal Growth. Executive functions showed moderate to small gains in Word Generation, Category Fluency, and Color-Word Switching. List Learning, List Recall, and Story Recall, also reflected cognitive gains at pre- and post-test for the resilience skills trained group in comparison to the wait list control group. The above findings are summarized in Figure 3 and Figure 4 below.

A particularly encouraging finding was the low attrition rate of 8% for the study sample, demonstrating that nearly all participants remained engaged in the resilience skills training program. This contrasts markedly with the high attrition rate of up to 40% for the most widely used treatment of direct exposure to traumatic experiences [138]. Both low attrition and post-test treatment effects provide evidence that the intervention operates as expected and benefits people suffering from PTSD.

### 3.2. Obesity

The results of the above clinical trial along with the early feasibility studies suggest that the GRIT intervention approach may be suitable for evocative, adverse contexts such as chronic pain and obesity. Certainly, the context in these cases is interoceptive and, in various degrees, also exteroceptive in representing complex biopsychosocial conditions. Both obesity and chronic pain represent national emergencies in the huge increase in obesity in the general population and among veterans [139]. Studying the resilience skills intervention with obesity provides an opportunity to test directly the efficacy of the goal-directed approach in calibrating and regulating the reward system, executive functions, and social relations. Preliminary cognitive findings (2013, 2012) of a subsample (*n* = 14): show higher scores in working memory for the control condition as reflected in Letter Number Sequencing (*p* = 0.631, partial eta squared = 0.01); higher scores for the treatment condition are: higher performance on measures of executive functions in List Learning (*p* = 0.346, partial eta squared = 0.042), Verbal Fluency Category Switching (*p* = 0.004, partial eta squared = 0.316), Verbal Fluency Category Switching Accuracy (*p* = 0.047, partial eta squared = 0.167), and Color-Word Interference Inhibition (*p* = 0.338, partial eta squared = 0.042). In sum, these findings indicate that the GRIT intervention enhances cognitive functioning in areas related to inhibitory control and short term episodic memory.

### 3.3. Chronic Pain

A clinical trial of the GRIT method of resilience skills training program is now under way for chronic pain, perhaps the most powerful aversive interoceptive context to which this approach may be adapted. The above-described design, manualized training with adaptation to pain, and outcome measures—including the addition of chronic pain scales—are adopted and observed in recruitment, pre-testing, and training. One additional component is the development of a short and long form of the program, to accommodate participants who may or may not have comorbid psychiatric conditions. The long form is designed to be completed over 8 sessions or 8 weeks. The short form extends over 4 sessions that are conducted during 2 weekly sessions for a total of 2 weeks.

## 4. Discussion

### 4.1. Innovations of the Goal-Directed Resilience Training Model

Scientists are often accused of operating in rather narrowly defined silos, rather oblivious to the relevant findings of scientists in adjacent fields. This trend has reversed to a great degree, and our work has embraced this movement towards interdisciplinary research. We view our trans-theoretical bridging of philosophy, neuroscience, psychological sciences, quantitative and qualitative methods in creating a framework for the Goal-Directed Resilience Training Model as an innovation. This holistic approach innovated by being at once theoretically grounded and person-centered. Growing out of this person-centered approach is our emphasis on goal-directed action in the GRIT intervention. By shifting focus away from the proverbial quantitative mean and paying closer attention to the qualitative experiences of exceptional survival at the tails of the continuum, we identified key ingredients for goal-directed resilience.

The duality of goal-directed action and stimulus-based responding has its roots in both proximal and distal origins. The distal origins reach at least to the classical Greek philosophers and their debates over the nature of a good life, followed by intermediary figures such as Jeremy Bentham, and the recent founders of scientific psychology headed by Darwin, William James, Freud, Kurt Lewin, and others. The proximal research literature is quite recent, covering the emergence of adult resilience studies over the past twenty years, the New Wave therapies that arose over this same twenty-year time period, and the emergence of this duality in various areas of contemporary neuroscience, cognition, affect, and other non-clinical psychological research areas. The novelty of the goal-directed resilience training described here is underscored when other investigators comment on the absence of research on goal-directed action in clinical and mental health research [68]. It is underscored when studies in neuroscience rather than in clinical research identify impairments in goal-directed action in psychiatric disorders [140,141,142,143]. The novelty of our approach is especially brought to the fore in the recognition that impaired goal-directed actions are common to various psychiatric conditions and could help in their classification [144,145]. The GRIT intervention identifies the loss of goal-directed action in clinical conditions and is a first attempt to address the loss and restore goal-directed action in a structured and well-articulated approach. The innovations we contribute to resilience and clinical research cover several areas:

(1) Theory and model. We identify goal-directed action as the main element that needs to be restored in chronic evocative contexts in which stimulus-based reactivity has resulted in maladaptive conditions such as PTSD or obesity. Our model posits a reciprocal relationship between prospective and reactive responding that accounts for both adaptive and maladaptive behaviors, rather than just focusing on a theory that accounts for symptoms.
(2) Method. Simulation is used in a step-by-step approach that includes re-experience of goal-directed elements that are then taken into a challenging situation, thereby simulating a resilient response. Our approach draws on personal experience and sensation to change bottom-up brain functions that have downstream effects that, in turn, help to restore top-down cognitive and affective processes through goal-directed action and positive affect, as are exemplified in engagement and social relatedness. The simulation includes repeated practice; special attention to sensation and expanding sensation beyond those felt in trauma, obesity, or pain; and special attention paid to the retention and generalization of gains.
(3) Efficacy. Multiple functions are assessed with a multi-method approach for testing efficacy. Effectiveness of the intervention assesses not only symptoms but also well-being, effects on cortical functions as assessed by neuropsychological measures, and effects on biological functions as assessed with endocrine measures of cortisol, leptin and adeponectin.
(4) Retention and generalization. Exercises allowed participants to apply concepts to their own lives, such as in “Life on Three Stages”. We sought to devise methods that incorporated the new knowledge gained from the intervention into participants’ lives. One approach was to have participants design a ‘good life’ with goals and a future they could look forward to, that could apply new learning that is generalized to other areas of their lives.

### 4.2. Limitations of the Goal-Directed Resilience Training Model

The innovations noted above also expose the limitations of this intervention.

(1) At the level of theory and model building, numerous details need to be developed. Examples are the adjustments and additional techniques in the intervention method for conditions that have unique characteristics beyond the evocative commonality, such as features unique to each of PTSD, obesity, pain, and others.
(2) Simulation can be enhanced by applications developed for new technologies.
(3) The multi-modal tests of efficacy do not yet include a wider array of endocrine functions and functional imaging. In addition, there is as yet no single scale that tests the main elements of the model, notably the predictability of the environment, approach-engagement, and social relatedness and how these three are affected by the intervention. So far we have used a patchwork of subscales from a variety of standardized tests, such as the SF-36 or Ryff’s Psychological Well-Being Scale. We are attempting to rectify this for chronic pain by developing a scale that will specifically test the main elements of the intervention.
(4) The extent to which gains are retained and generalized need to be evaluated over time.

Among the limitations we should acknowledge are that we have completed only limited pilot testing and, though the data is encouraging, more controlled testing needs to be done with all three evocative context groups: trauma, obesity, and chronic pain. Our veteran population is not generalizable to the rest of the population due to their unique experiences. Further, we do not exclude people because of comorbid illnesses or require that they refrain from other treatment while going through the intervention. Therefore, there is a possibility that effects we detect are due to other causes. This is, however, a common limitation with patient samples.

### 4.3. Future Directions of Goal-Directed Resilience Training

A productive direction for this work will be to apply this model to areas that have been particularly difficult to treat or that place various groups at risk: (1) dual diagnoses such as chronic pain, obesity, chronic illness and mental health diagnoses; (2) professions that conduct their work in highly evocative contexts, such as the police; (3) minority populations whose status evokes prejudice and who are at risk for less favorable treatment in main areas of their lives, of education, employment, or interacting with the justice system.

All three evocative context groups will require reasonably sized controlled intervention trials in veteran and non-veteran groups in order to collect more data on the program’s efficacy. Like all intervention researchers, we analyze our and reanalyze our choice of measures, constructs, study populations, environments, and other moving parts for improvements. As a result, for the GRIT- chronic pain intervention we are developing a chronic pain diary and scale that uniquely emphasizes goal-directed resilience aspects of chronic pain, not currently captured by pain scales. While the psychometric challenges of validating a new scale are daunting, our investigative team has committed to the process. Other challenges facing the GRIT intervention include those of expanding the program to additional populations, such as experiences of inner city trauma and an innovative intervention program initiated in collaboration with our program and Dr. Wrenn at Morehouse School of Medicine [146]. In addition, we seek to develop research designs that help to identify the most useful and powerful elements of the program and to follow up longitudinally to determine the extent of the program’s effectiveness.

### 4.4. Conclusion

Resilience skills training restores native capacities for goal-directed responding in the face of adversity and breaks the cycle of chronic self-maintaining, maladaptive conditions. The remarkable survival we detected in narratives of individuals adapting well in extreme conditions reveal capacities that can simulated through resilience skills training. To be sure, the Gulag and the Hanoi Hilton are not equivalent to PTSD symptoms in the free United States of America, the unstoppable taste of raspberry-topped crème brulée ice cream, or unrelieved chronic low back pain. But the stimuli in all three examples are persistent and demand a response, be they startle responses to a sudden stimulus resembling past threat, loss of inhibitory control in obesity, or a persistent quest to ameliorate pain. Resilience skills training employs simulations in which these conditions recede into the background and goal-directed actions take center-stage in the person’s life. Russo and colleagues [147] concluded that, “it should be possible to induce natural mechanisms of resilience” [147] (p. 1482). The goal-directed resilience training program activates natural mechanisms of resilience and helps participants to apply them to their own challenges.

There is much truth in Baldwin’s [148] statement that, “Not everything that is faced can be changed, but nothing can be changed until it is faced”. And, we would add, change depends on *how* we face things. The goal-directed resilience training approach teaches participants to face challenges through displacing reactivity to inexorable contexts with goals and plans of engagement that restore a more complete and richer life.
